# Assessing *ST18* gene polymorphisms (rs17315309, rs2304365) in Iraqi patients with Pemphigus vulgaris

**DOI:** 10.25122/jml-2023-0227

**Published:** 2024-01

**Authors:** Zahra Ali Al-Hasnawi, Ban AL-Drobie

**Affiliations:** 1 Department of Oral Medicine, College of Dentistry, University of Baghdad, Baghdad, Iraq; 2 Department of Oral Diagnosis, College of Dentistry, University of Baghdad, Baghdad, Iraq

**Keywords:** Pemphigus vulgaris, rs17315309, rs2304365, *ST18* gene, PV, Pemphigus Vulgaris, S, Significant, NS, Non-significant, HS, Highly Significant, NMC, Normalized Melting Curves, DC, Differential Curves, MAF, Minor Allele Frequency

## Abstract

Pemphigus vulgaris (PV) is a potentially fatal autoimmune disease characterized by blistering of the skin, mucous membranes, and oral cavity. Genetics are implicated in its etiology, with the *ST18* gene identified as a potential risk factor for pemphigus in certain populations, suggesting its role as a novel molecular target for therapeutic intervention. This study aimed to detect single nucleotide polymorphisms (SNPs) rs17315309 A/G and rs2304365 C/G in the *ST18* gene among Iraqi/Arabic patients with PV. A total of 90 Iraqi subjects participated in this study, including 45 patients diagnosed with PV and 45 healthy controls. SNP analysis was performed using High-Resolution Melt Analysis (HRMA) with Eva Green I Dye. For SNP rs17315309 A/G, the distribution of heterozygous genotypes showed highly significant differences between the patient and healthy groups (*P* = 0.005), with the mutant G-allele being significantly more prevalent in patients than in the healthy group (*P* = 0.001). In contrast, for SNP rs2304365 C/G, the distribution of heterozygous and mutant genotypes did not differ significantly between patients and healthy individuals (*P* = 0.8 and *P* = 0.3, respectively), with the mutant G-allele also showing no significant difference (*P* = 0.4). Our data indicate a significant association between PV and the rs17315309 A/G SNP in the *ST18* gene among the Iraqi population of Arabic origin. However, no association was found between patients with PV and the rs2304365 C/G SNP in the same gene.

## INTRODUCTION

Pemphigus vulgaris (PV) is a severe autoimmune blistering disease that primarily affects the skin and mucous membranes, including the oral cavity. Genetics is believed to play a significant role in its development. The *ST18* gene has emerged as a potential contributor, potentially increasing the risk of PV in certain populations, and could represent a new potential molecular target for the treatment of disease. PV is a relatively rare disease, affecting approximately 0.07 to 1.6 per 100,000 people annually, with uneven geographic distribution [1]. It has been associated with a 2.3-3.3-fold increased fatality rate compared to the general population, primarily due to complications such as pneumonia or sepsis [2]. This disease is characterized by the production of specific autoantibodies against desmogleins 1 and 3. These antibodies disrupt the adhesion between keratinocytes (skin cells), leading to acantholysis and, ultimately, blister formation. PV is diagnosed clinically and by histopathological biopsy, which may be done on the skin or oral mucosa. Biopsies of affected skin or oral mucosa are often taken for two types of analysis: direct immunofluorescence (DIF) to detect the presence of autoantibodies and histology to examine tissue samples under a microscope for characteristic features of PV [3]. Oral lesions often appear before skin involvement, affecting 80-90% of patients with PV at some point. These lesions may exist for weeks or months before the skin lesions appear. PV may sometimes affect only the oral mucosa in certain people. These lesions can manifest as shallow, irregular erosions on the inner cheeks (buccal mucosa), palate, tongue, and gums (gingiva). As the lesions heal, they may develop a more defined border and a whitish appearance before disappearing completely [4].

Growing evidence suggests a genetic influence on PV development. While the disease can affect individuals of any age or gender, epidemiological data reveals variations across populations. For instance, PV appears more prevalent in Caucasians and East Asians, while pemphigus foliaceus, another form, is more common in South America and North Africa. Age and gender predilection also appear to vary across regions, suggesting the combined influence of genetics, hormones, and environmental factors on PV and its subtypes [5]. While the human leukocyte antigen (HLA) system is known to be involved in autoimmune diseases, recent research has focused on the roles of non-HLA genes in PV. Candidate gene studies have explored potential associations between genes related to the immune system and PV, in particular autoantigens, cytokines, and immunoglobulins, such as tumor necrosis factor-alpha (TNF-α), interleukin-6 (IL-6), IL-10, desmoglein 3 (DSG3), VH3, *TAP2, ST18*, CD86, and *NR3C1* [6].

Genome-wide association studies (GWAS) have provided further insights into the genetic basis of PV. A study by Sarig *et al*. employing GWAS in the Jewish population identified an association between PV and polymorphisms (variations) within the *ST18* gene on chromosome 8q11.23 [7]. The *ST18* gene, known for its role in suppressing tumorigenicity, is involved in apoptosis and inflammation regulation, encoding a zinc-finger DNA-binding protein. These functions are crucial in the pathophysiology of PV, acting through mechanisms associated with a transcription factor directly implicated in the disease [8]. Further research highlighted that a single nucleotide polymorphism (SNP) in the *ST18* promoter region, associated with PV, appears to increase gene transcription activity. Experimental evidence has shown that *ST18* is activated by PV serum, leading to acantholysis (the breakdown of cellular adhesion in the skin) and the production of key inflammatory mediators. These findings underscore the direct involvement of *ST18* in the pathogenesis of PV [9,10]. To our knowledge, this is the first study that aimed to determine the relationship between *ST18* gene polymorphisms (rs17315309 A/G and rs2304365 C/G) and the incidence of Pemphigus vulgaris within the Iraqi population.

## MATERIAL AND METHODS

### Study design and population

This case-control study was conducted between April and November 2022 at the Department of Dermatology, Baghdad Teaching Hospital. We included a cohort of 90 individuals from Central Iraq, including 45 patients with clinically and histopathologically confirmed PV and 45 healthy control individuals without any history of autoimmune disorders, cancer, infectious diseases, systemic inflammation, or allergies. None of the controls were related to the patients with PV. The groups were matched for age and gender.

### Blood collection

Approximately two milliliters of venous blood were collected from each participant by trained healthcare professionals. The blood was immediately transferred to tubes containing Ethylenediaminetetraacetic Acid (EDTA) and stored at low temperatures in a deep freezer [11,12].

### SNPs selection

We focused on the *ST18* gene located on chromosome 8q11.23 for SNP selection. We identified SNPs by reviewing previously published studies, specifically those involving the *ST18* gene. Additionally, we utilized the database of Single Nucleotide Polymorphisms (dbSNP), which is a part of the National Center for Biotechnology Information (NCBI) [13].

### Primer design

Primers for amplifying the selected SNPs were designed using the Primer3 Plus web service and cross-validated by the University Code of Student Conduct programs. Reference sequences were obtained from the NCBI database. Primers were synthesized and supplied in a lyophilized form by Alpha DNA (Table 1).

**Table 1:** Primer sequences for *ST18* gene SNPs

Primer	Sequence (5'→3' direction)	Primer Size (bp)
*ST18* (rs17315309)		
Forward	5’-CTTCTCGTGATTAGCATACAAA-3’	22
Reverse	5’-GCCAGGCAGCAATTCATTTC-3’	20
*ST18* (rs2304365)		
Forward	5’AGCTTTCCAAATTCAACCCAAGA-3’	23
Reverse	5’-GCAAAACTTGGAGGAAAACATGC-3’	23

### DNA extraction and analysis

Genomic DNA was extracted from the stored frozen blood samples using the Easy Pure Genomic DNA Kit (Transgene Biotech). The extraction process was conducted in strict accordance with the manufacturer's recommended protocol [14]. The concentration and purity of the extracted DNA were assessed using a NanoDrop spectrophotometer. Agarose gel electrophoresis was then employed to confirm the presence and quality of the isolated genomic DNA (gDNA) as well as the PCR amplification products, ensuring their suitability for further analysis [15,16].

### High-Resolution Melting (HRM) analysis for genotyping

We investigated the association between the selected *ST18* gene SNPs (rs17315309 A/G and rs2304365 C/G) and PV in Iraqi patients using real-time PCR with HRM analysis. The Trans Start Tip Green qPCR Super Mix (Trans Gen Biotech, AQ141-01) and Eva Green I Dye were employed.

### HRM real-time PCR technique

The Rotor-Gene Q Real-Time PCR System (QIAGEN) was used for qPCR-HRM analysis. The HRM analysis involved a temperature gradient from 55°C to 95°C with an increment of 0.2°C. Duplicate reactions were performed using synthetic SNP sequences with 2xTransStart Tip Green qPCR Super Mix. Triplicate synthetic controls were subjected to qPCR-HRM to identify allelic variations. Normalized melting curves (NMC) and differential melting curves (DC) were generated using the HRM software integrated into the Rotor-Gene 4.4 software.

### Statistical analysis

Data analysis was performed using the Rotor-Gene Q Series Software and included recording Ct values, amplification plots, dissociation curves, HRM curves, and a consolidated report. Further statistical analyses were conducted using SPSS for Windows, version 26. Descriptive statistics were expressed as mean ± standard deviation (SD). Genotype and allele frequencies were evaluated for Hardy-Weinberg equilibrium (HWE) [17]. A Chi-square test (χ^2^ test) was used to compare frequencies between patient and control groups, with a *P* value of less than 0.05 considered statistically significant.

## RESULTS

This study recruited a total of 90 participants from Central Iraq. The study groups comprised 45 patients diagnosed with PV and 45 healthy controls with no history of relevant medical conditions. The socio-demographic characteristics, including age and sex distribution, are presented in Table 2. There was no significant difference in age and sex distribution between the two groups, suggesting that any deviations observed in the patient group could be attributed to the effects of PV rather than demographic disparities.

**Table 2:** Distribution of age and sex across groups

Variables		PV group				Control		*P* value
			*n*	%		*n*	%	
Age	20–30		5	11.1		9	20	χ^2^ = 9.394 *P* = 0.052 (NS)
	30–40		6	13.3		15	33.3	
	40–50		19	42.2		15	33.3	
	50–60		9	20.0		4	8.9	
	60–70		6	13.3		2	4.4	
	Total		45	100		45	100	
	Mean ± SD		45.36 ± 10.60			37.02 ± 10.49		
	Range		27–67			21–70		
Gender	Men		14		31.1	14	31.1	χ^2^ = 0.000 *P* = 1.000 (NS)
	Women		31		68.9	31	68.9	
	Total		45		100	45	100	
C.S. (*) *P*-value			χ^2^ = 3.411 *P* = 0.492 (NS)			χ^2^ = 3.007 *P* = 0.557 (NS)		

C.S. (*), Computer statistic; NS, Non-Sig. at P >0.05; Testing based on one and two samples Chi-Square tests (χ^2^); SD, standard deviation, n, number.

### Genotype and allele frequency for SNPs rs17315309A/G and rs2304365C/G

Table 3 shows the genotype frequencies of SNPs rs17315309 and rs2304365 within the *ST18* gene. Both SNPs were confirmed to be in Hardy-Weinberg equilibrium (*P* >0.05). For the SNP rs17315309, the hetero genotype distributions between patients and the control group showed considerable variations (OR = 0.21; 95% CI, 0. 0746–0.6414; *P* <0.005) and major variations in the distributions of mutant genotypes (OR = 0.09l; 95% CI, 0.0279–0.3393; *P* <0.003) (A/G). The patient group had considerably higher mutant G-allele frequencies than the control group (OR = 0.2; 95% CI, 0.1399–0.4839; *P* <0.001). For the SNP rs2304365, there were non-significant variations in the hetero genotype distributions between patients and control (OR = 0.9; 95% CI, 0.3638–2.2642; *P* <0.8), and non-significant differences in the mutant genotype distributions (OR = 1.7; 95% CI, 0.5263–5.9172; *P* <0.3) (C/G), and allele frequencies for the mutant G-allele (OR = 1.2; 95%CI, 0.6934–2.2976; *P* <0.4,).

**Table 3:** Genotype and allele frequencies of the 2 SNPs in the *ST18* gene

SNP		Type	PV group (*n* = 45)	Control group (*n* = 45)	*P* value	OR	95% CI
rs17315309 A/G	Genotype frequencies	AA (wild)	24 (53.3%)	7 (15.5%)	--	1.00	(Reference)
		AG (hetero)	15 (33.3%)	20 (44.4%)	0.005**	0.21	0.0746–0.6414
		GG (mutant)	6 (13.3%)	18 (40%)	0.003*	0.09	0.0279–0.3393
	Allele frequencies	A (wild)	63 (70.00%)	34 (37.78%)	--	1.00	(Reference)
		G (mutant)	27 (30.00%)	56 (62.22%)	0.001**	0.2	0.1399–0.4839
		Number	n = 90	n = 90			
rs2304365 C/G	Genotype frequencies	CC (wild)	17 (37.8%)	18 (40%)	--	1.00	(Reference)
		CG (hetero)	18 (40%)	21 (46.6%)	0.8	0.9	0.3638–2.2642
		GG (mutant)	10 (22.2%)	6 (13.3%)	0.3	1.7	0.5263–5.9172
	Allele frequencies	C (wild)	52 (57.78%)	57 (63.33%)	--	1.00	(Reference)
		G (mutant)	38 (42.22%)	33 (36.67%)	0.4	1.2	0.6934–2.2976
		Number	*n* = 90	*n* = 90			

*P* value (*P* ≤0.05), ** highly significant; OR, Odds ratio; 95%CI. 95 percent confidence interval.

### Observed and expected allele frequencies

Table 4 shows the genotype distributions for SNPs rs17315309A/G and rs2304365C/G and the observed and expected allele frequencies in both the patient and control groups. This table also includes the allele frequencies anticipated under the Hardy-Weinberg Equilibrium and examines the significance of these frequencies between the groups studied. The chi-squared (χ^2^) test for HWE showed no significant difference from the expected frequencies in either patient or control groups (*P* > 0.05).

**Table 4:** Genotype distribution and allele frequencies for SNPs rs17315309 A/G and rs2304365 C/G

Symbol	Genotype	Patients group			Control group		
		Obs.	Exp.	H-W Freq.	Obs.	Exp.	H-W Freq.
rs17315309 A/G	Wild AA	24	22.05	9%	7	6.42	14.2%
	Hetero AG	15	18.9	42%	20	21.16	47.01%
	Mutant GG	6	4.05	49%	18	17.42	38.72%
H-W Equilibrium χ^2^ value at 5%		*P* = 0.1663 NS			*P* = 0.7141 NS		
rs2304365 C/G	Wild C/C	17	15.02	33.38%	18	18.05	40.1%
	Hetero C/G	18	21.96	48.79%	21	20.90	46.4%
	Mutant G/G	10	8.02	17.83%	6	6.05	13.44%
H-W Equilibrium χ^2^ value at 5%		*P* = 0.2268 NS			*P* = 0.9744 NS		

χ^2^ ≥ 3.841 Sig. at *P* <0.05; NS: Non-Sig. at *P* ≥0.05; The null hypothesis is that the population is at H-W equilibrium; Obs., observed; Exp., expected; G, guanine; C, cytosine.

## DISCUSSION

Our findings revealed a potential correlation between the rs17315309 polymorphism within the *ST18* gene and PV risk in the Iraqi population. This is the first study to investigate this association in an Iraqi cohort, highlighting the potential for rs17315309 as a novel target for future research. However, limitations include the relatively small sample size due to the rarity of PV and the need to explore additional SNPs within the *ST18* gene.

In terms of age demographics, the mean age of our patient group was 45.36 ± 10.60 years, supporting findings from previous studies conducted in Iraq (average age 46 years) [18] and India (average age 41.3 ± 13.65 years) [19]. Conversely, studies from Poland reported an average age range of 50 to 55 years (51.6 years) [20], while Japanese research indicated an average patient age of 62 years [21]. The results could be due to a difference in study sample size and ethnic variations. Regarding gender distribution, most patients in the PV and control groups were women (68.9%), and only 31.1% were men. There was no statistical difference in gender distribution (χ^2^ = 0.000 *P* = 1.000). This finding supports the commonly observed trend of higher susceptibility to PV in female subjects, aligning with studies from Iraq [4] and Slovakia [22]. The female prevalence of this autoimmune disease can be explained by hormonal (estrogen, progesterone), immunological, and genetic (specific genes encoded on the X chromosome) factors, which are known to modulate autoimmune responses, particularly during periods of hormonal fluctuation like pregnancy and postpartum. The estrogen hormone may play a major role in increasing PV incidence among females since this hormone has been reported to enhance immunological reactions [23].

Our investigation revealed a significant association between SNP rs17315309 in the *ST18* gene and PV among the Iraqi population, supporting the findings of Vodo *et al*. [9] from the 2016 Tel-Aviv study. They reported that rs17315309 significantly increased the risk for PV within the Jewish community, a finding that aligns with our observations. This SNP was discovered to promote higher gene transcription in a p53/p63-dependent manner, which might explain why *ST18* is upregulated in PV patients' skin [9]. Our results are further supported by Assaf *et al*., who identified an overexpression of the *ST18* gene associated with rs17315309 in the skin of patients with PV [24]. This overexpression is implicated in the release of inflammatory cytokines and PV IgG-induced acantholysis of keratinocytes, with a particular emphasis on the role of TNFα. Elevated levels of TNFα, previously detected in the serum of patients with PV, may exacerbate the disease, suggesting that increased TNFα production could act as a pathway for the detrimental effects of *ST18* in PV pathogenesis [24]. However, this result disagrees with another study, which included the Italian Caucasian population and observed an increased presence of the risk allele in control subjects compared to PV patients for rs17315309. This highlights the complex interplay of genetic factors and suggests potential ethnic-specific influences on the disease's genetic predisposition [10].

The genotype and allele frequencies for SNP rs2304365 presented in our study did not show a significant relationship with PV in the Iraqi cohort. This finding aligns with previous studies. For instance, a study by Sarig *et al*. observed no significant association between this SNP and PV in a Jewish population of German descent [7]. Similarly, research by Yue *et al*. [25] found no relationship between rs2304365 and PV in a Han Chinese population. These findings suggest that the contribution of *ST18*-related polymorphisms to PV susceptibility may vary between populations, reinforcing the concept that genetic risk factors for PV can be highly population-specific.

Minor allele frequency (MAF) analysis of rs2304365 revealed significant variations across populations: African (0.402), European (0.208), and Han Chinese (0.119) from Beijing. The significant difference in MAF between African and other populations may point towards a unique evolutionary trajectory, potentially explaining why a connection was observed only within the African cohort but not among German or Chinese descent. [25]. Additionally, this contrasts with a study from Tehran by Etesami *et al*., which found a significant correlation between SNP rs2304365 and PV in the Iranian population. Their results suggested that the risk allele might lead to an overexpression of *ST18*, prompting keratinocytes to release cytokines. This process could facilitate the onset of pemphigus by increasing the vulnerability of keratinocytes to IgG-mediated cell-cell detachment [11].

## CONCLUSION

This study identified an association between PV and the SNPs rs17315309 A/G within the *ST18* gene among the Iraqi population of Arab origin. However, there was no association between PV and the SNPs rs2304365 C/G. To completely understand the relevance of the *ST18* gene in Iraqi patients with PV, more replication studies within varied genetic backgrounds are required.
